# Bis[3-(2*H*-benzotriazol-2-yl)-2-(prop-2-yn­yloxy)-5-(2,4,4-trimethyl­pentan-2-yl)phen­yl]methane

**DOI:** 10.1107/S1600536811006374

**Published:** 2011-02-26

**Authors:** Tahir Qadri, Itrat Anis, M. R. Shah, Seik Weng Ng

**Affiliations:** a H.E.J. Research Institute of Chemistry, International Center for Chemical and Biological Sciences, University of Karachi, Karachi 7527, Pakistan; b Department of Chemistry, University of Malaya, 50603 Kuala Lumpur, Malaysia

## Abstract

In the title compound, C_47_H_54_N_6_O_2_, the C—C—C bond angle between the rings is  108.40 (13)°. One aryl ring aligned at 38.5 (1)° with respect to the *N*-heterocyclic substituent and the other at 56.0 (1)° with respect to its substituent. In the crystal, adjacent mol­ecules are linked by C—H⋯N hydrogen bonds, forming a chain extending along the *a* axis.

## Related literature

For a similar compound, see: Ali *et al.* (2011 ▶).
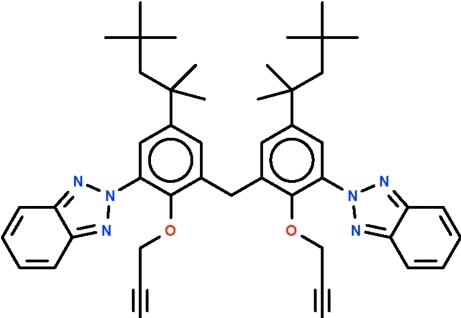


## Experimental

### 

#### Crystal data

C_47_H_54_N_6_O_2_
*M* *_r_* = 734.96
Triclinic, 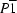

*a* = 11.4805 (4) Å
*b* = 13.8247 (4) Å
*c* = 14.6180 (6) Å
α = 104.808 (3)°
β = 103.706 (3)°
γ = 100.642 (3)°
*V* = 2103.58 (14) Å^3^
*Z* = 2
Mo *K*α radiation
μ = 0.07 mm^−1^
*T* = 100 K
0.30 × 0.25 × 0.20 mm

#### Data collection

Agilent SuperNova Dual diffractometer with an Atlas detector
Absorption correction: multi-scan (*CrysAlis PRO*; Agilent, 2010 ▶) *T*_min_ = 0.822, *T*_max_ = 1.000
17578 measured reflections
9290 independent reflections
6994 reflections with *I* > 2σ(*I*)
*R*_int_ = 0.031

#### Refinement

*R*[*F* ^2^ > 2σ(*F* ^2^)] = 0.051
*wR*(*F* ^2^) = 0.127
*S* = 1.01
9290 reflections
514 parameters
2 restraints
H atoms treated by a mixture of independent and constrained refinement
Δρ_max_ = 0.47 e Å^−3^
Δρ_min_ = −0.30 e Å^−3^

### 

Data collection: *CrysAlis PRO* (Agilent, 2010 ▶); cell refinement: *CrysAlis PRO*; data reduction: *CrysAlis PRO*; program(s) used to solve structure: *SHELXS97* (Sheldrick, 2008 ▶); program(s) used to refine structure: *SHELXL97* (Sheldrick, 2008 ▶); molecular graphics: *X-SEED* (Barbour, 2001 ▶); software used to prepare material for publication: *publCIF* (Westrip, 2010 ▶).

## Supplementary Material

Crystal structure: contains datablocks global, I. DOI: 10.1107/S1600536811006374/zs2092sup1.cif

Structure factors: contains datablocks I. DOI: 10.1107/S1600536811006374/zs2092Isup2.hkl

Additional supplementary materials:  crystallographic information; 3D view; checkCIF report

## Figures and Tables

**Table 1:** Hydrogen-bond geometry (Å, °)

*D*—H⋯*A*	*D*—H	H⋯*A*	*D*⋯*A*	*D*—H⋯*A*
C41—H41⋯N3^i^	0.96 (1)	2.38 (1)	3.283 (3)	158 (2)

Symmetry code: (i) 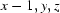.

**Fig. 1.**
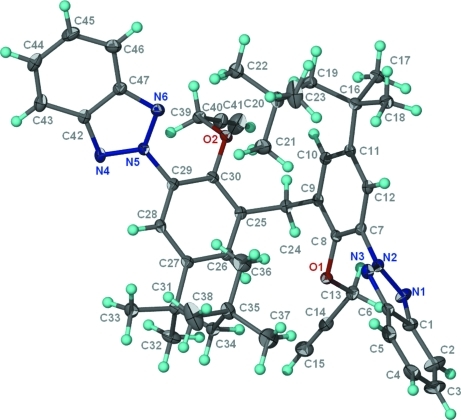
Thermal ellipsoid plot (Barbour, 2001) of C47H54N6O2 at the 70% probability level with hydrogen atoms drawn as spheres of arbitrary radius.

## supplementary crystallographic information

### Comment

Some background on di(aryl)methane compounds having oxyacetate substituents was presented in an earlier report (Ali *et al.*, 2011). The title compound also has an *N*-heterocyclic substituent in the rings (Scheme I).

### Experimental

6,6'-Methylenebis(2-(2*H*-benzo[*d*][1,2,3]triazol-2-yl)-4-(2,4,4-\ trimethylpentan-2-yl)phenol) (0.01 g) and potassium carbonate (0.05 g) were dissolved in acetone (20 ml) at 323 K. Propargyl bromide (0.04 ml) was added and the reaction was stirred for 20 h. The progress of the reaction was monitored by thin layer chromatography (hexane: dichloromethane 60:40). The reaction was quenched by adding 1 M hydrochloric acid (10 ml). The aqueous phase was extracted with dichloromethane, the solvent evaporated and the crude product was recrystallized from dichloromethane (yield 80%).

### Refinement

Carbon-bound H-atoms were placed in calculated positions [C—H = 0.95 to 0.99 Å and *U*_iso_(H) = 1.2 to 1.5*U*_eq_(C)] and were included in the refinement in the riding model approximation. The acetylenic H-atoms were located in a difference Fourier map and were refined with a distance restraint of C—H = 0.95±0.01 Å and their isotropic displacement parameters also refining. The structure contains solvent accessible voids of 66 Å^3^.

### Crystal data

**Table:**

C_47_H_54_N_6_O_2_	*Z* = 2
*M**_r_* = 734.96	*F*(000) = 788
Triclinic, *P*1	*D*_x_ = 1.160 Mg m^−^^3^
Hall symbol: -P 1	Mo *K*α radiation, λ = 0.71073 Å
*a* = 11.4805 (4) Å	Cell parameters from 6715 reflections
*b* = 13.8247 (4) Å	θ = 2.5–29.3°
*c* = 14.6180 (6) Å	µ = 0.07 mm^−^^1^
α = 104.808 (3)°	*T* = 100 K
β = 103.706 (3)°	Block, beige
γ = 100.642 (3)°	0.30 × 0.25 × 0.20 mm
*V* = 2103.58 (14) Å^3^	

### Data collection

**Table:**

Agilent SuperNova Dual diffractometer with an Atlas detector	9290 independent reflections
Radiation source: SuperNova (Mo) X-ray Source	6994 reflections with *I* > 2σ(*I*)
Mirror	*R*_int_ = 0.031
Detector resolution: 10.4041 pixels mm^-1^	θ_max_ = 27.5°, θ_min_ = 2.5°
ω scans	*h* = −11→14
Absorption correction: multi-scan (*CrysAlis PRO*; Agilent, 2010)	*k* = −17→17
*T*_min_ = 0.822, *T*_max_ = 1.000	*l* = −18→18
17578 measured reflections	

### Refinement

**Table:**

Refinement on *F*^2^	Primary atom site location: structure-invariant direct methods
Least-squares matrix: full	Secondary atom site location: difference Fourier map
*R*[*F*^2^ > 2σ(*F*^2^)] = 0.051	Hydrogen site location: inferred from neighbouring sites
*wR*(*F*^2^) = 0.127	H atoms treated by a mixture of independent and constrained refinement
*S* = 1.01	*w* = 1/[σ^2^(*F*_o_^2^) + (0.0421*P*)^2^ + 1.0461*P*] where *P* = (*F*_o_^2^ + 2*F*_c_^2^)/3
9290 reflections	(Δ/σ)_max_ = 0.001
514 parameters	Δρ_max_ = 0.47 e Å^−^^3^
2 restraints	Δρ_min_ = −0.30 e Å^−^^3^

### Fractional atomic coordinates and isotropic or equivalent isotropic displacement parameters (Å^2^)

**Table:**

	*x*	*y*	*z*	*U*_iso_*/*U*_eq_	
O1	0.62069 (10)	0.57849 (8)	0.68366 (8)	0.0181 (2)	
O2	0.46427 (10)	0.92915 (9)	0.74295 (8)	0.0180 (2)	
N1	0.75682 (14)	0.48200 (11)	0.55515 (11)	0.0253 (3)	
N2	0.79630 (13)	0.58497 (10)	0.58114 (10)	0.0175 (3)	
N3	0.91866 (13)	0.62735 (11)	0.61580 (10)	0.0210 (3)	
N4	0.63903 (13)	1.12608 (10)	1.02490 (10)	0.0191 (3)	
N5	0.62242 (12)	1.07152 (10)	0.93082 (9)	0.0170 (3)	
N6	0.62881 (13)	1.12402 (11)	0.86637 (10)	0.0208 (3)	
C1	0.86471 (17)	0.45397 (14)	0.57584 (13)	0.0243 (4)	
C2	0.8856 (2)	0.35465 (16)	0.56490 (17)	0.0382 (5)	
H2	0.8190	0.2935	0.5382	0.046*	
C3	1.0059 (2)	0.35097 (17)	0.59449 (17)	0.0418 (6)	
H3	1.0231	0.2854	0.5892	0.050*	
C4	1.1068 (2)	0.44130 (18)	0.63295 (14)	0.0368 (5)	
H4	1.1891	0.4345	0.6529	0.044*	
C5	1.08845 (18)	0.53777 (16)	0.64196 (13)	0.0313 (4)	
H5	1.1561	0.5981	0.6666	0.038*	
C6	0.96427 (16)	0.54370 (14)	0.61302 (12)	0.0218 (4)	
C7	0.71225 (15)	0.64916 (12)	0.57571 (11)	0.0164 (3)	
C8	0.62335 (15)	0.64286 (12)	0.62555 (11)	0.0160 (3)	
C9	0.54520 (14)	0.70952 (12)	0.62302 (11)	0.0159 (3)	
C10	0.55881 (15)	0.77843 (12)	0.56992 (11)	0.0169 (3)	
H10	0.5046	0.8226	0.5672	0.020*	
C11	0.64859 (15)	0.78633 (12)	0.52001 (11)	0.0165 (3)	
C12	0.72566 (15)	0.71951 (12)	0.52388 (11)	0.0175 (3)	
H12	0.7876	0.7222	0.4909	0.021*	
C13	0.52191 (16)	0.48396 (12)	0.63901 (12)	0.0214 (4)	
H13A	0.5508	0.4290	0.5995	0.026*	
H13B	0.4500	0.4956	0.5944	0.026*	
C14	0.48590 (16)	0.45259 (13)	0.71871 (13)	0.0227 (4)	
C15	0.45817 (19)	0.42907 (15)	0.78391 (15)	0.0319 (4)	
C16	0.66093 (15)	0.86743 (13)	0.46638 (12)	0.0187 (3)	
C17	0.53433 (16)	0.84807 (14)	0.38963 (12)	0.0229 (4)	
H17A	0.5128	0.7787	0.3416	0.034*	
H17B	0.5391	0.9001	0.3550	0.034*	
H17C	0.4705	0.8531	0.4235	0.034*	
C18	0.75675 (17)	0.85806 (14)	0.40902 (13)	0.0237 (4)	
H18A	0.7281	0.7915	0.3558	0.036*	
H18B	0.8367	0.8619	0.4543	0.036*	
H18C	0.7665	0.9147	0.3803	0.036*	
C19	0.68883 (15)	0.97889 (12)	0.53917 (12)	0.0193 (3)	
H19A	0.6861	1.0258	0.4981	0.023*	
H19B	0.6176	0.9802	0.5661	0.023*	
C20	0.80758 (16)	1.03051 (13)	0.62902 (13)	0.0227 (4)	
C21	0.8235 (2)	0.97002 (15)	0.70266 (14)	0.0368 (5)	
H21A	0.8414	0.9048	0.6723	0.055*	
H21B	0.7469	0.9552	0.7210	0.055*	
H21C	0.8924	1.0112	0.7621	0.055*	
C22	0.79155 (19)	1.13578 (14)	0.68323 (14)	0.0295 (4)	
H22A	0.8666	1.1738	0.7387	0.044*	
H22B	0.7200	1.1248	0.7084	0.044*	
H22C	0.7779	1.1759	0.6371	0.044*	
C23	0.92515 (19)	1.05220 (19)	0.59749 (16)	0.0462 (6)	
H23A	0.9497	0.9877	0.5772	0.069*	
H23B	0.9923	1.1028	0.6533	0.069*	
H23C	0.9088	1.0800	0.5418	0.069*	
C24	0.45816 (15)	0.71523 (12)	0.68700 (11)	0.0173 (3)	
H24A	0.3871	0.7399	0.6573	0.021*	
H24B	0.4254	0.6457	0.6914	0.021*	
C25	0.53039 (14)	0.78978 (12)	0.78966 (11)	0.0163 (3)	
C26	0.59913 (15)	0.75508 (12)	0.86006 (12)	0.0172 (3)	
H26	0.5928	0.6829	0.8451	0.021*	
C27	0.67730 (15)	0.82160 (12)	0.95195 (12)	0.0171 (3)	
C28	0.68230 (15)	0.92638 (12)	0.97296 (12)	0.0171 (3)	
H28	0.7345	0.9739	1.0348	0.020*	
C29	0.61161 (15)	0.96223 (12)	0.90424 (12)	0.0165 (3)	
C30	0.53422 (14)	0.89489 (12)	0.81258 (11)	0.0160 (3)	
C31	0.75537 (15)	0.77823 (13)	1.02367 (12)	0.0191 (3)	
C32	0.66482 (18)	0.70338 (15)	1.05332 (14)	0.0299 (4)	
H32A	0.6130	0.7411	1.0849	0.045*	
H32B	0.7122	0.6746	1.0999	0.045*	
H32C	0.6118	0.6469	0.9940	0.045*	
C33	0.83766 (17)	0.86281 (14)	1.11939 (13)	0.0266 (4)	
H33A	0.7852	0.8932	1.1563	0.040*	
H33B	0.8890	0.9169	1.1030	0.040*	
H33C	0.8914	0.8325	1.1602	0.040*	
C34	0.83234 (16)	0.71225 (13)	0.97307 (13)	0.0229 (4)	
H34A	0.8710	0.6800	1.0214	0.027*	
H34B	0.7716	0.6550	0.9169	0.027*	
C35	0.93593 (17)	0.75639 (15)	0.93242 (13)	0.0274 (4)	
C36	0.89336 (18)	0.81043 (16)	0.85695 (15)	0.0335 (5)	
H36A	0.9604	0.8295	0.8289	0.050*	
H36B	0.8719	0.8731	0.8896	0.050*	
H36C	0.8203	0.7636	0.8038	0.050*	
C37	0.9750 (2)	0.66217 (19)	0.87945 (17)	0.0488 (6)	
H37A	1.0385	0.6850	0.8495	0.073*	
H37B	0.9026	0.6127	0.8276	0.073*	
H37C	1.0088	0.6288	0.9275	0.073*	
C38	1.05044 (19)	0.8306 (2)	1.01414 (16)	0.0518 (7)	
H38A	1.1175	0.8475	0.9854	0.078*	
H38B	1.0775	0.7976	1.0644	0.078*	
H38C	1.0297	0.8943	1.0449	0.078*	
C39	0.36827 (15)	0.97167 (13)	0.77280 (12)	0.0194 (3)	
H39A	0.3669	1.0357	0.7545	0.023*	
H39B	0.3869	0.9901	0.8457	0.023*	
C40	0.24673 (17)	0.89734 (14)	0.72536 (13)	0.0255 (4)	
C41	0.14749 (19)	0.83803 (16)	0.68885 (17)	0.0388 (5)	
C42	0.65798 (15)	1.22441 (13)	1.02168 (12)	0.0190 (3)	
C43	0.68485 (16)	1.31885 (13)	1.09851 (13)	0.0252 (4)	
H43	0.6879	1.3205	1.1644	0.030*	
C44	0.70612 (17)	1.40743 (14)	1.07339 (14)	0.0297 (4)	
H44	0.7256	1.4723	1.1235	0.036*	
C45	0.70014 (18)	1.40630 (14)	0.97485 (15)	0.0314 (4)	
H45	0.7158	1.4702	0.9612	0.038*	
C46	0.67257 (18)	1.31596 (14)	0.89937 (14)	0.0287 (4)	
H46	0.6677	1.3155	0.8335	0.034*	
C47	0.65172 (15)	1.22327 (13)	0.92390 (12)	0.0206 (4)	
H15	0.438 (2)	0.4082 (16)	0.8366 (12)	0.047 (6)*	
H41	0.0684 (14)	0.7883 (15)	0.6599 (17)	0.060 (8)*	

### Atomic displacement parameters (Å^2^)

**Table:**

	*U*^11^	*U*^22^	*U*^33^	*U*^12^	*U*^13^	*U*^23^
O1	0.0199 (6)	0.0156 (5)	0.0173 (6)	0.0022 (5)	0.0042 (5)	0.0055 (4)
O2	0.0175 (6)	0.0234 (6)	0.0157 (5)	0.0079 (5)	0.0059 (4)	0.0077 (5)
N1	0.0278 (8)	0.0169 (7)	0.0367 (9)	0.0076 (6)	0.0168 (7)	0.0098 (6)
N2	0.0173 (7)	0.0162 (7)	0.0190 (7)	0.0043 (6)	0.0067 (5)	0.0045 (5)
N3	0.0170 (7)	0.0241 (7)	0.0194 (7)	0.0066 (6)	0.0041 (6)	0.0032 (6)
N4	0.0181 (7)	0.0212 (7)	0.0163 (7)	0.0043 (6)	0.0068 (6)	0.0020 (6)
N5	0.0179 (7)	0.0175 (7)	0.0151 (7)	0.0041 (6)	0.0052 (5)	0.0045 (5)
N6	0.0226 (8)	0.0192 (7)	0.0204 (7)	0.0046 (6)	0.0052 (6)	0.0076 (6)
C1	0.0298 (10)	0.0250 (9)	0.0294 (9)	0.0139 (8)	0.0182 (8)	0.0143 (8)
C2	0.0448 (13)	0.0301 (10)	0.0611 (14)	0.0200 (10)	0.0348 (11)	0.0254 (10)
C3	0.0586 (15)	0.0444 (13)	0.0558 (14)	0.0372 (12)	0.0393 (12)	0.0345 (11)
C4	0.0404 (12)	0.0596 (14)	0.0274 (10)	0.0361 (11)	0.0160 (9)	0.0205 (10)
C5	0.0271 (10)	0.0448 (12)	0.0214 (9)	0.0187 (9)	0.0047 (8)	0.0053 (8)
C6	0.0249 (9)	0.0289 (9)	0.0161 (8)	0.0137 (8)	0.0079 (7)	0.0082 (7)
C7	0.0148 (8)	0.0148 (7)	0.0169 (8)	0.0038 (6)	0.0029 (6)	0.0021 (6)
C8	0.0162 (8)	0.0162 (8)	0.0123 (7)	0.0017 (6)	0.0019 (6)	0.0032 (6)
C9	0.0125 (8)	0.0162 (8)	0.0143 (7)	0.0010 (6)	0.0017 (6)	0.0010 (6)
C10	0.0146 (8)	0.0178 (8)	0.0165 (8)	0.0045 (6)	0.0030 (6)	0.0034 (6)
C11	0.0156 (8)	0.0170 (8)	0.0144 (7)	0.0029 (6)	0.0027 (6)	0.0033 (6)
C12	0.0167 (8)	0.0186 (8)	0.0159 (8)	0.0037 (7)	0.0060 (6)	0.0029 (6)
C13	0.0230 (9)	0.0166 (8)	0.0213 (8)	0.0000 (7)	0.0069 (7)	0.0037 (7)
C14	0.0218 (9)	0.0188 (8)	0.0277 (9)	0.0055 (7)	0.0079 (7)	0.0069 (7)
C15	0.0341 (11)	0.0338 (11)	0.0361 (11)	0.0095 (9)	0.0170 (9)	0.0187 (9)
C16	0.0185 (8)	0.0212 (8)	0.0185 (8)	0.0066 (7)	0.0062 (6)	0.0081 (7)
C17	0.0230 (9)	0.0247 (9)	0.0211 (8)	0.0074 (7)	0.0043 (7)	0.0087 (7)
C18	0.0275 (10)	0.0266 (9)	0.0222 (9)	0.0096 (8)	0.0119 (7)	0.0106 (7)
C19	0.0201 (9)	0.0193 (8)	0.0217 (8)	0.0061 (7)	0.0082 (7)	0.0090 (7)
C20	0.0196 (9)	0.0252 (9)	0.0216 (9)	0.0035 (7)	0.0076 (7)	0.0047 (7)
C21	0.0439 (13)	0.0253 (10)	0.0276 (10)	0.0054 (9)	−0.0077 (9)	0.0050 (8)
C22	0.0342 (11)	0.0227 (9)	0.0264 (9)	0.0031 (8)	0.0053 (8)	0.0055 (8)
C23	0.0221 (11)	0.0617 (15)	0.0357 (11)	−0.0067 (10)	0.0102 (9)	−0.0058 (11)
C24	0.0142 (8)	0.0182 (8)	0.0183 (8)	0.0024 (6)	0.0051 (6)	0.0051 (6)
C25	0.0139 (8)	0.0190 (8)	0.0173 (8)	0.0033 (6)	0.0077 (6)	0.0056 (6)
C26	0.0167 (8)	0.0170 (8)	0.0193 (8)	0.0047 (7)	0.0079 (6)	0.0056 (6)
C27	0.0156 (8)	0.0212 (8)	0.0173 (8)	0.0054 (7)	0.0078 (6)	0.0077 (7)
C28	0.0158 (8)	0.0199 (8)	0.0153 (8)	0.0036 (7)	0.0062 (6)	0.0046 (6)
C29	0.0152 (8)	0.0160 (8)	0.0188 (8)	0.0036 (6)	0.0073 (6)	0.0045 (6)
C30	0.0136 (8)	0.0218 (8)	0.0158 (8)	0.0069 (7)	0.0065 (6)	0.0079 (6)
C31	0.0177 (8)	0.0218 (8)	0.0197 (8)	0.0066 (7)	0.0068 (7)	0.0078 (7)
C32	0.0269 (10)	0.0400 (11)	0.0321 (10)	0.0116 (9)	0.0109 (8)	0.0233 (9)
C33	0.0268 (10)	0.0315 (10)	0.0213 (9)	0.0142 (8)	0.0040 (7)	0.0061 (8)
C34	0.0238 (9)	0.0244 (9)	0.0207 (8)	0.0099 (7)	0.0044 (7)	0.0069 (7)
C35	0.0212 (9)	0.0398 (11)	0.0226 (9)	0.0124 (8)	0.0077 (7)	0.0077 (8)
C36	0.0260 (10)	0.0461 (12)	0.0358 (11)	0.0118 (9)	0.0164 (8)	0.0168 (9)
C37	0.0559 (15)	0.0667 (16)	0.0456 (13)	0.0417 (13)	0.0297 (12)	0.0225 (12)
C38	0.0215 (11)	0.0903 (19)	0.0316 (11)	0.0005 (12)	0.0081 (9)	0.0089 (12)
C39	0.0173 (8)	0.0222 (8)	0.0218 (8)	0.0093 (7)	0.0073 (7)	0.0075 (7)
C40	0.0214 (10)	0.0267 (9)	0.0279 (9)	0.0080 (8)	0.0074 (7)	0.0067 (8)
C41	0.0235 (11)	0.0349 (11)	0.0492 (13)	0.0047 (9)	0.0092 (9)	0.0021 (10)
C42	0.0139 (8)	0.0204 (8)	0.0214 (8)	0.0045 (7)	0.0055 (6)	0.0044 (7)
C43	0.0209 (9)	0.0248 (9)	0.0265 (9)	0.0055 (7)	0.0093 (7)	0.0005 (7)
C44	0.0264 (10)	0.0205 (9)	0.0355 (10)	0.0057 (8)	0.0079 (8)	−0.0010 (8)
C45	0.0337 (11)	0.0189 (9)	0.0400 (11)	0.0068 (8)	0.0069 (9)	0.0101 (8)
C46	0.0335 (11)	0.0245 (9)	0.0286 (10)	0.0076 (8)	0.0057 (8)	0.0123 (8)
C47	0.0174 (8)	0.0199 (8)	0.0232 (8)	0.0058 (7)	0.0036 (7)	0.0061 (7)

### Geometric parameters (Å, °)

**Table:**

O1—C8	1.3795 (19)	C22—H22A	0.9800
O1—C13	1.4442 (19)	C22—H22B	0.9800
O2—C30	1.3801 (19)	C22—H22C	0.9800
O2—C39	1.4446 (19)	C23—H23A	0.9800
N1—N2	1.3342 (19)	C23—H23B	0.9800
N1—C1	1.355 (2)	C23—H23C	0.9800
N2—N3	1.3343 (19)	C24—C25	1.519 (2)
N2—C7	1.429 (2)	C24—H24A	0.9900
N3—C6	1.350 (2)	C24—H24B	0.9900
N4—N5	1.3375 (18)	C25—C26	1.385 (2)
N4—C42	1.351 (2)	C25—C30	1.395 (2)
N5—N6	1.3350 (19)	C26—C27	1.396 (2)
N5—C29	1.434 (2)	C26—H26	0.9500
N6—C47	1.353 (2)	C27—C28	1.389 (2)
C1—C6	1.406 (3)	C27—C31	1.529 (2)
C1—C2	1.413 (3)	C28—C29	1.389 (2)
C2—C3	1.361 (3)	C28—H28	0.9500
C2—H2	0.9500	C29—C30	1.394 (2)
C3—C4	1.420 (3)	C31—C33	1.532 (2)
C3—H3	0.9500	C31—C32	1.544 (2)
C4—C5	1.367 (3)	C31—C34	1.556 (2)
C4—H4	0.9500	C32—H32A	0.9800
C5—C6	1.412 (2)	C32—H32B	0.9800
C5—H5	0.9500	C32—H32C	0.9800
C7—C12	1.386 (2)	C33—H33A	0.9800
C7—C8	1.390 (2)	C33—H33B	0.9800
C8—C9	1.401 (2)	C33—H33C	0.9800
C9—C10	1.384 (2)	C34—C35	1.547 (3)
C9—C24	1.521 (2)	C34—H34A	0.9900
C10—C11	1.401 (2)	C34—H34B	0.9900
C10—H10	0.9500	C35—C36	1.521 (3)
C11—C12	1.394 (2)	C35—C38	1.530 (3)
C11—C16	1.528 (2)	C35—C37	1.537 (3)
C12—H12	0.9500	C36—H36A	0.9800
C13—C14	1.462 (2)	C36—H36B	0.9800
C13—H13A	0.9900	C36—H36C	0.9800
C13—H13B	0.9900	C37—H37A	0.9800
C14—C15	1.175 (3)	C37—H37B	0.9800
C15—H15	0.951 (19)	C37—H37C	0.9800
C16—C18	1.538 (2)	C38—H38A	0.9800
C16—C17	1.540 (2)	C38—H38B	0.9800
C16—C19	1.558 (2)	C38—H38C	0.9800
C17—H17A	0.9800	C39—C40	1.460 (2)
C17—H17B	0.9800	C39—H39A	0.9900
C17—H17C	0.9800	C39—H39B	0.9900
C18—H18A	0.9800	C40—C41	1.183 (3)
C18—H18B	0.9800	C41—H41	0.956 (10)
C18—H18C	0.9800	C42—C47	1.410 (2)
C19—C20	1.551 (2)	C42—C43	1.414 (2)
C19—H19A	0.9900	C43—C44	1.362 (3)
C19—H19B	0.9900	C43—H43	0.9500
C20—C21	1.521 (3)	C44—C45	1.422 (3)
C20—C23	1.530 (3)	C44—H44	0.9500
C20—C22	1.536 (2)	C45—C46	1.364 (3)
C21—H21A	0.9800	C45—H45	0.9500
C21—H21B	0.9800	C46—C47	1.411 (2)
C21—H21C	0.9800	C46—H46	0.9500
			
C8—O1—C13	113.93 (12)	C20—C23—H23C	109.5
C30—O2—C39	114.29 (12)	H23A—C23—H23C	109.5
N2—N1—C1	102.29 (14)	H23B—C23—H23C	109.5
N3—N2—N1	117.53 (14)	C25—C24—C9	108.40 (13)
N3—N2—C7	120.33 (13)	C25—C24—H24A	110.0
N1—N2—C7	122.11 (13)	C9—C24—H24A	110.0
N2—N3—C6	102.41 (13)	C25—C24—H24B	110.0
N5—N4—C42	102.34 (13)	C9—C24—H24B	110.0
N6—N5—N4	117.52 (13)	H24A—C24—H24B	108.4
N6—N5—C29	122.02 (12)	C26—C25—C30	119.10 (14)
N4—N5—C29	120.13 (13)	C26—C25—C24	120.18 (14)
N5—N6—C47	102.38 (13)	C30—C25—C24	120.62 (15)
N1—C1—C6	108.80 (15)	C25—C26—C27	122.80 (15)
N1—C1—C2	130.06 (18)	C25—C26—H26	118.6
C6—C1—C2	121.14 (18)	C27—C26—H26	118.6
C3—C2—C1	116.6 (2)	C28—C27—C26	117.44 (15)
C3—C2—H2	121.7	C28—C27—C31	122.76 (14)
C1—C2—H2	121.7	C26—C27—C31	119.78 (14)
C2—C3—C4	122.51 (19)	C29—C28—C27	120.53 (15)
C2—C3—H3	118.7	C29—C28—H28	119.7
C4—C3—H3	118.7	C27—C28—H28	119.7
C5—C4—C3	121.61 (19)	C28—C29—C30	121.39 (15)
C5—C4—H4	119.2	C28—C29—N5	117.37 (14)
C3—C4—H4	119.2	C30—C29—N5	121.24 (15)
C4—C5—C6	116.91 (19)	O2—C30—C29	122.25 (14)
C4—C5—H5	121.5	O2—C30—C25	119.07 (14)
C6—C5—H5	121.5	C29—C30—C25	118.66 (15)
N3—C6—C1	108.96 (15)	C27—C31—C33	112.41 (14)
N3—C6—C5	129.86 (17)	C27—C31—C32	107.48 (14)
C1—C6—C5	121.17 (17)	C33—C31—C32	107.02 (14)
C12—C7—C8	121.83 (15)	C27—C31—C34	111.73 (13)
C12—C7—N2	118.48 (14)	C33—C31—C34	111.62 (14)
C8—C7—N2	119.63 (14)	C32—C31—C34	106.16 (14)
O1—C8—C7	119.86 (14)	C31—C32—H32A	109.5
O1—C8—C9	120.97 (14)	C31—C32—H32B	109.5
C7—C8—C9	118.90 (15)	H32A—C32—H32B	109.5
C10—C9—C8	118.53 (14)	C31—C32—H32C	109.5
C10—C9—C24	120.36 (14)	H32A—C32—H32C	109.5
C8—C9—C24	120.71 (14)	H32B—C32—H32C	109.5
C9—C10—C11	123.25 (15)	C31—C33—H33A	109.5
C9—C10—H10	118.4	C31—C33—H33B	109.5
C11—C10—H10	118.4	H33A—C33—H33B	109.5
C12—C11—C10	117.22 (15)	C31—C33—H33C	109.5
C12—C11—C16	122.74 (14)	H33A—C33—H33C	109.5
C10—C11—C16	120.02 (14)	H33B—C33—H33C	109.5
C7—C12—C11	120.26 (15)	C35—C34—C31	123.94 (15)
C7—C12—H12	119.9	C35—C34—H34A	106.3
C11—C12—H12	119.9	C31—C34—H34A	106.3
O1—C13—C14	107.94 (13)	C35—C34—H34B	106.3
O1—C13—H13A	110.1	C31—C34—H34B	106.3
C14—C13—H13A	110.1	H34A—C34—H34B	106.4
O1—C13—H13B	110.1	C36—C35—C38	108.52 (18)
C14—C13—H13B	110.1	C36—C35—C37	107.67 (16)
H13A—C13—H13B	108.4	C38—C35—C37	107.99 (18)
C15—C14—C13	178.8 (2)	C36—C35—C34	113.78 (15)
C14—C15—H15	177.3 (14)	C38—C35—C34	113.00 (15)
C11—C16—C18	112.27 (14)	C37—C35—C34	105.57 (17)
C11—C16—C17	107.74 (13)	C35—C36—H36A	109.5
C18—C16—C17	107.06 (14)	C35—C36—H36B	109.5
C11—C16—C19	111.17 (13)	H36A—C36—H36B	109.5
C18—C16—C19	111.89 (13)	C35—C36—H36C	109.5
C17—C16—C19	106.36 (13)	H36A—C36—H36C	109.5
C16—C17—H17A	109.5	H36B—C36—H36C	109.5
C16—C17—H17B	109.5	C35—C37—H37A	109.5
H17A—C17—H17B	109.5	C35—C37—H37B	109.5
C16—C17—H17C	109.5	H37A—C37—H37B	109.5
H17A—C17—H17C	109.5	C35—C37—H37C	109.5
H17B—C17—H17C	109.5	H37A—C37—H37C	109.5
C16—C18—H18A	109.5	H37B—C37—H37C	109.5
C16—C18—H18B	109.5	C35—C38—H38A	109.5
H18A—C18—H18B	109.5	C35—C38—H38B	109.5
C16—C18—H18C	109.5	H38A—C38—H38B	109.5
H18A—C18—H18C	109.5	C35—C38—H38C	109.5
H18B—C18—H18C	109.5	H38A—C38—H38C	109.5
C20—C19—C16	123.93 (14)	H38B—C38—H38C	109.5
C20—C19—H19A	106.3	O2—C39—C40	110.99 (13)
C16—C19—H19A	106.3	O2—C39—H39A	109.4
C20—C19—H19B	106.3	C40—C39—H39A	109.4
C16—C19—H19B	106.3	O2—C39—H39B	109.4
H19A—C19—H19B	106.4	C40—C39—H39B	109.4
C21—C20—C23	110.31 (18)	H39A—C39—H39B	108.0
C21—C20—C22	107.26 (15)	C41—C40—C39	178.5 (2)
C23—C20—C22	107.14 (16)	C40—C41—H41	178.0 (16)
C21—C20—C19	113.27 (15)	N4—C42—C47	108.93 (14)
C23—C20—C19	112.49 (15)	N4—C42—C43	130.09 (16)
C22—C20—C19	105.95 (15)	C47—C42—C43	120.94 (16)
C20—C21—H21A	109.5	C44—C43—C42	116.72 (17)
C20—C21—H21B	109.5	C44—C43—H43	121.6
H21A—C21—H21B	109.5	C42—C43—H43	121.6
C20—C21—H21C	109.5	C43—C44—C45	122.34 (16)
H21A—C21—H21C	109.5	C43—C44—H44	118.8
H21B—C21—H21C	109.5	C45—C44—H44	118.8
C20—C22—H22A	109.5	C46—C45—C44	121.85 (18)
C20—C22—H22B	109.5	C46—C45—H45	119.1
H22A—C22—H22B	109.5	C44—C45—H45	119.1
C20—C22—H22C	109.5	C45—C46—C47	116.80 (18)
H22A—C22—H22C	109.5	C45—C46—H46	121.6
H22B—C22—H22C	109.5	C47—C46—H46	121.6
C20—C23—H23A	109.5	N6—C47—C42	108.82 (15)
C20—C23—H23B	109.5	N6—C47—C46	129.79 (16)
H23A—C23—H23B	109.5	C42—C47—C46	121.34 (15)
			
C1—N1—N2—N3	0.61 (18)	C16—C19—C20—C23	67.1 (2)
C1—N1—N2—C7	−177.38 (14)	C16—C19—C20—C22	−176.18 (15)
N1—N2—N3—C6	−0.50 (18)	C10—C9—C24—C25	−88.59 (17)
C7—N2—N3—C6	177.52 (14)	C8—C9—C24—C25	84.04 (17)
C42—N4—N5—N6	0.14 (18)	C9—C24—C25—C26	−85.49 (18)
C42—N4—N5—C29	173.68 (14)	C9—C24—C25—C30	90.85 (17)
N4—N5—N6—C47	−0.13 (18)	C30—C25—C26—C27	−3.1 (2)
C29—N5—N6—C47	−173.53 (14)	C24—C25—C26—C27	173.28 (14)
N2—N1—C1—C6	−0.44 (18)	C25—C26—C27—C28	1.7 (2)
N2—N1—C1—C2	179.75 (19)	C25—C26—C27—C31	−176.83 (14)
N1—C1—C2—C3	−178.54 (19)	C26—C27—C28—C29	0.0 (2)
C6—C1—C2—C3	1.7 (3)	C31—C27—C28—C29	178.46 (14)
C1—C2—C3—C4	−1.1 (3)	C27—C28—C29—C30	−0.2 (2)
C2—C3—C4—C5	−0.4 (3)	C27—C28—C29—N5	−179.48 (14)
C3—C4—C5—C6	1.2 (3)	N6—N5—C29—C28	138.04 (15)
N2—N3—C6—C1	0.17 (17)	N4—N5—C29—C28	−35.2 (2)
N2—N3—C6—C5	−178.86 (17)	N6—N5—C29—C30	−41.3 (2)
N1—C1—C6—N3	0.2 (2)	N4—N5—C29—C30	145.51 (15)
C2—C1—C6—N3	−179.99 (16)	C39—O2—C30—C29	−65.62 (19)
N1—C1—C6—C5	179.31 (15)	C39—O2—C30—C25	116.27 (15)
C2—C1—C6—C5	−0.9 (3)	C28—C29—C30—O2	−179.32 (14)
C4—C5—C6—N3	178.34 (17)	N5—C29—C30—O2	0.0 (2)
C4—C5—C6—C1	−0.6 (3)	C28—C29—C30—C25	−1.2 (2)
N3—N2—C7—C12	55.3 (2)	N5—C29—C30—C25	178.07 (14)
N1—N2—C7—C12	−126.81 (16)	C26—C25—C30—O2	−179.05 (13)
N3—N2—C7—C8	−122.00 (16)	C24—C25—C30—O2	4.6 (2)
N1—N2—C7—C8	55.9 (2)	C26—C25—C30—C29	2.8 (2)
C13—O1—C8—C7	−104.28 (16)	C24—C25—C30—C29	−173.61 (14)
C13—O1—C8—C9	81.71 (17)	C28—C27—C31—C33	0.1 (2)
C12—C7—C8—O1	−174.05 (13)	C26—C27—C31—C33	178.55 (15)
N2—C7—C8—O1	3.1 (2)	C28—C27—C31—C32	117.60 (17)
C12—C7—C8—C9	0.1 (2)	C26—C27—C31—C32	−63.94 (19)
N2—C7—C8—C9	177.24 (13)	C28—C27—C31—C34	−126.31 (16)
O1—C8—C9—C10	174.77 (13)	C26—C27—C31—C34	52.1 (2)
C7—C8—C9—C10	0.7 (2)	C27—C31—C34—C35	63.8 (2)
O1—C8—C9—C24	2.0 (2)	C33—C31—C34—C35	−63.1 (2)
C7—C8—C9—C24	−172.06 (14)	C32—C31—C34—C35	−179.34 (16)
C8—C9—C10—C11	−1.3 (2)	C31—C34—C35—C36	−56.0 (2)
C24—C9—C10—C11	171.51 (14)	C31—C34—C35—C38	68.3 (2)
C9—C10—C11—C12	1.0 (2)	C31—C34—C35—C37	−173.84 (17)
C9—C10—C11—C16	−177.59 (14)	C30—O2—C39—C40	−102.14 (16)
C8—C7—C12—C11	−0.4 (2)	N5—N4—C42—C47	−0.09 (17)
N2—C7—C12—C11	−177.54 (14)	N5—N4—C42—C43	−177.86 (17)
C10—C11—C12—C7	−0.2 (2)	N4—C42—C43—C44	176.48 (17)
C16—C11—C12—C7	178.39 (14)	C47—C42—C43—C44	−1.1 (2)
C8—O1—C13—C14	−149.87 (14)	C42—C43—C44—C45	0.9 (3)
C12—C11—C16—C18	6.2 (2)	C43—C44—C45—C46	0.0 (3)
C10—C11—C16—C18	−175.24 (14)	C44—C45—C46—C47	−0.8 (3)
C12—C11—C16—C17	123.87 (16)	N5—N6—C47—C42	0.06 (17)
C10—C11—C16—C17	−57.60 (18)	N5—N6—C47—C46	177.52 (18)
C12—C11—C16—C19	−119.96 (16)	N4—C42—C47—N6	0.02 (19)
C10—C11—C16—C19	58.56 (19)	C43—C42—C47—N6	178.03 (15)
C11—C16—C19—C20	62.8 (2)	N4—C42—C47—C46	−177.70 (16)
C18—C16—C19—C20	−63.7 (2)	C43—C42—C47—C46	0.3 (3)
C17—C16—C19—C20	179.77 (15)	C45—C46—C47—N6	−176.57 (18)
C16—C19—C20—C21	−58.9 (2)	C45—C46—C47—C42	0.6 (3)

### Hydrogen-bond geometry (Å, °)

**Table:**

*D*—H···*A*	*D*—H	H···*A*	*D*···*A*	*D*—H···*A*
C41—H41···N3^i^	0.96 (1)	2.38 (1)	3.283 (3)	158 (2)

Symmetry codes: (i) *x*−1, *y*, *z*.
